# The need for kidney biopsy in the management of side effects of target and immunotherapy

**DOI:** 10.3389/fneph.2023.1043874

**Published:** 2023-02-27

**Authors:** Roberta Fenoglio, Martina Cozzi, Giulio Del Vecchio, Savino Sciascia, Antonella Barreca, Alessandro Comandone, Dario Roccatello

**Affiliations:** ^1^ CMID-Nephrology and Dialysis Unit (ERK-net, ERN-Reconnect and RITA-ERN Member), San Giovanni Bosco Hub Hospital and Department of Clinical and Biological Sciences, University of Torino, Turin, Italy; ^2^ Division of Pathology, Città della Salute e della Scienza Hospital and University of Turin, Turin, Italy; ^3^ Division of Oncology, San Giovanni Bosco Hub Hospital, Turin, Italy

**Keywords:** onconephrology, immune checkpoint inhibitors, targeted therapies, renal biopsy, renale adverse events of anticancer treatments, thrombothic micro-angiopathy (TMA), interstitial tubular nephritis

## Abstract

**Introduction:**

The introduction of innovative therapies, resulting from revisiting cancer as a disease of the immune system, has changed the scenario of complications. These new classes of drugs, such as targeted therapies and immune checkpoint inhibitors, assure substantial advantages in cancer therapy, despite some side effects affecting various organs, including the kidney. Histological evaluations of kidney disorders induced by targeted/immunotherapy are limited.

**Method:**

In this study we examined the histological features of patients treated with new cancer agents who underwent a kidney biopsy for new onset kidney failure and/or urinary abnormalities.

**Results:**

The cohort included 30 adult patients. The most frequently administered therapies were immunotherapy (30%), targeted therapy (26.7%), immunotherapy plus targeted therapy (13.3%), immunotherapy plus chemotherapy (13.3%), targeted therapy plus chemotherapy (16.7%). The most common histological finding was tubular interstitial nephritis (30%) that was associated with acute tubular necrosis in 4 cases, and thrombotic microangiopathy (23.3%). After kidney biopsy, 16 of the 30 patients were treated according to the histological diagnosis. Fourteen patients were treated with steroids. One patient with membranous nephropathy was treated with a single dose of rituximab. A patient with severe thrombotic microangiopathy requiring dialysis received a treatment with eculizumab for 3 months. Overall some renal response was obtained in all patients treated with glucocorticoids, while complete kidney response was achieved in the patient treated with rituximab. Cancer treatment was resumed without change in 21 out of 30 patients.

**Conclusion:**

Kidney biopsy is critical for the management of kidney toxicities and should be strongly encouraged for patients showing adverse kidney effects of novel cancer agents.

## Introduction

Over time, nephrologists have learned about the most frequent side effects related to the use of conventional drugs such as cisplatin or alkylating agents (1, 2). However, in recent years the introduction of innovative therapies, resulting from revisiting oncologic disease as a disease of the immune system, has changed the scenario of complications (3).

After a few years from the introduction of these new agents it became clear that kidney was one of the targeted organs for possible toxicity (4). An initial delay in the recognition of kidney adverse effects was due to the timing of kidney damage development, that is later than the observation period of registration studies (5) and the exclusion of patients with known kidney impairment from registration studies.

Kidney disease has a significant impact on the management of cancer patients leading to either temporary or definitive discontinuation of therapy, prescription of inadequate doses, and use of suboptimal drugs. All these promote tumor growth and metastases.

Anti-angiogenic drugs are responsible for vascular and/or glomerular damage. The spectrum of the possible histological lesions is constantly evolving, and besides thrombotic microangiopathy (TMA) and focal segmental glomerulosclerosis (FSGS), others histological features are emerging (6).

Among the targeted drugs, tyrosine kinase inhibitors (TKIs) are taking a growing role. TKIs are involved in the signal transduction pathways that regulates cell proliferation, differentiation, migration, metabolism and anti-apoptotic signals. The signal pathway that is most used by TKIs, as some monoclonal antibodies, is that of vascular endothelial growth factor A - vascular endothelial growth factor receptor 2 (VEGFA-VEGFR2) (7).

A review of 72 randomized controlled trials showed a significant association between the use of TKIs and the appearance of proteinuria as a consequence of a podocytopathy related to an altered phosphorylation of nephrine (8). Accordingly, histological findings of minimal change disease (MCD) and FSGS have been reported (9–11).

For instance, recent reports suggest that gefitinib, an epidermal growth factor receptor (EGFR) inhibitor, can trigger diverse renal kidney pathologies including MCD, tubulo-interstitial nephritis (TIN), membranous nephropathy (MN) and IgA nephropathy (IgAN) (12–14).

Immune checkpoint inhibitors (ICIs) are monoclonal antibodies that counteract tumor-induced repression of T lymphocytes by interacting with tumor cell ligands and inhibiting their interaction with T lymphocytes. Acute kidney injury (AKI) (15) and proteinuria have been reported.

However, to establish incidence and define the type of kidney damage histologic evidence is needed (16–21).

In addition to their antiangiogenic activity, inhibitors of the vascular endothelial growth factor (VEGF) pathway enhance the anti-cancer activity of ICIs by blocking tumor-induced immune-suppressive cells and increasing T-cell infiltration into tumors (22–24). Based on this evidence, both the Food and Drug Administration (FDA) and the European Medicines Agency (EMA) recently approved axitinib, a tyrosine kinase inhibitor, in combination with avelumab or pembrolizumab, anti-programmed cell death protein 1/ligand 1 (PD-1/PD-L1), for the first-line treatment of patients with advanced kidney cell carcinoma (25–27).

The use of ICIs in combination with antiangiogenic agents renders both the diagnosis and the management of toxicities more complex The use of ICIs in combination with antiangiogenic agents renders both the diagnosis and the management of toxicities more complex, as some of the adverse events (AEs) of ICIs are similar to antiangiogenic therapy. Recognizing the specific etiology of each AE based on a histologic basis is critical to optimize the continuation of treatment (28) and strategies to distinguish between immune-related AEs caused by ICIs versus those resulting from TKIs are needed to optimize the management of side effects due to multi drug therapy.

The recommendations for kidney biopsy in patients with cancer have recently been updated (29). However, indications remain restricted. Kidney biopsy has been recommended in patients who present with new onset proteinuria (≥1 g per day) or worsening kidney function when the diagnosis of kidney disease cannot be otherwise established (30). This report aims at dispelling the myth that kidney biopsy must be limited to these restricted clinical settings. In order to address this scope we reviewed the histological features of patients treated with new cancer therapies (i.e., targeted therapies and immunotherapies) who underwent a kidney biopsy for new onset kidney impairment and/or urinary abnormalities.

## Materials and methods

This is a single-center retrospective observational study on patients with cancer treated with targeted/immunotherapy who underwent kidney biopsy of the native kidney between July 2017 and July 2022. Laboratory and clinical data were evaluated at the time of kidney biopsy. Demographic data, medication history, type of cancer, comorbidities, diagnosis of kidney disease and therapeutic decisions were collected. Kidney function was assessed by measuring estimated glomerular filtration rate, in accordance with the Kidney Disease Outcomes Quality Initiative guidelines using the formula of Modification of Diet in kidney Disease, and proteinuria obtained by 24-hour urine collection. AKI was defined according to the Kidney Disease Improving Global Outcomes (KDIGO) criteria. Biopsy indication for AKI required at least a doubling of serum creatinine (sCr) values from those at baseline. Histological evaluation was performed by a single pathologist.

## Results

Our cohort included 30 adult patients (16 males and 14 females) with ongoing targeted/immuno- therapies. Demographic and clinical characteristics are shown in **Table 1**. The mean age was 65 years (range 32-82) at the time of biopsy. The most frequent sites of malignancies were the lung (23.3%), the urinary tract (20%) and the abdomen (13.3%). Six patients had 2 different cancers. Overall, 73.3% of patients had stage IV cancer or higher.

**Table 1:** Demographic and clinical characteristics, treatment at the time of biopsy and biopsy indications.

Pt	Sex	Age	Cancer	Previous treatment	Treatment at the time of Biopsy	Baseline sCr mg/dl	Biopsy Indication
1	M	72	Lung	No	NKTR-214 + nivolumab + carboplatin + pemetrexed	0.7	AKI
2	F	59	Breast	No	letrolo e ribociclib	0.6	AKI + uPT
3	M	71	Kidney	Yes (1)	nivolumab	1.3	AKI + U Abn
4	F	58	Ovary	Yes (1)	bevacizumab + carboplatin + paclitaxel	0.8	uPT
5	F	65	Breast	Yes (2)	beva+Taxotere+RT+tamoxi/taxol avastin	0.9	AKI + uPT
6	M	62	Lung	No	pembrolizumab + pemetrexed + cisplatin	0.7	RPGN
7	M	79	Bladder+Lung	Yes (1)	nivolumab	0.8	AKI + uPT
8	F	68	Myelofibrosis	No	ruxolitinib	1.3	uPT
9	F	56	Sarcoma	Yes (3)	sunitinib e nivolumab	0.6	uPT
10	M	64	Lung	No	pembrolizumab	1	AKI + uPT
11	M	50	Lung	No	pemetrexed+ pembrolizumab	1.3	AKI
12	M	73	Bowel	Yes (1)	alibercept + folfirinox	0.8	uPT
13	M	82	Liver	No	sorefenib	1.7	AKI + uPT
14	F	72	Kidney	No	pembrolizumab + axitinib	0.6	AKI
15	F	68	Bowel	No	bevacizumab + capecitabina	0.7	uPT
16	M	61	Larynx	Yes (1)	pembrolizumab	0.8	AKI
17	F	52	Lung	Yes (2)	pembrolizumab + cisplatin + pemetrexed	0.8	AKI
18	F	66	Lung	Yes (1)	nivolumab	0.8	U Abn
19	M	47	Non Hodg Lym	Yes (1)	ibrutinib	1.3	AKI
20	M	80	Bladder + Melanoma	No	pembrolizumab	1.8	AKI
21	M	59	Bowel	No	bevacizumab	0.8	uPT
22	F	49	Ovary	Yes (1)	bevacizumab	0.7	uPT
23	F	67	Lung	No	sunitinib	1.3	AKI
24	M	71	kidney	Yes (1)	pembrolizumab + axitinib	1.2	AKI + U Abn
25	M	62	Lung + Bladder	Yes (1)	nivolumab	1.7	AKI
26	F	53	Sarcoma	Yes (>3)	nivolumab + sunitinib	0.7	AKI + uPT
27	M	71	Larynx+Kidney	Yes (1)	nivolumab	1.2	AKI + U Abn
28	M	72	Larynx + Lung	Yes (1)	nivolumab	1.5	AKI + uPT
29	F	67	Breast	Yes (1)	ribociclib	0.7	AKI + U Abn
30	F	75	Breast + GIST	Yes (1)	sunitinib	1	uPT

AKI, acute kidney injury; uPT, proteinuria; U Abn, urinary abnormalities; RPGN, rapidly progressive glomerulonephritis; Beva, bevacizumab; tamoxi, tamoxifen.

**Figure 1:**
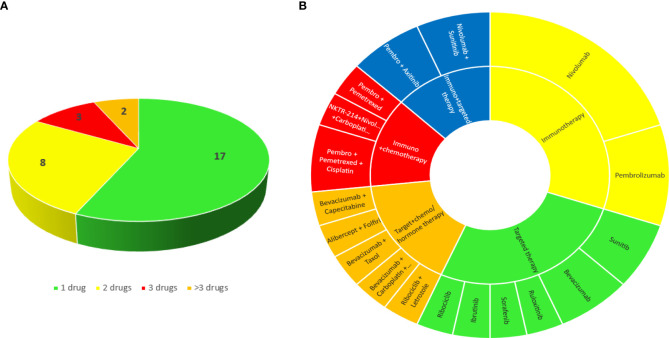
Number **(A)** and classes **(B)** of cancer drugs that patients were taking at the time of the biopsy.

Oncologic treatments are summarized in **Figure 1**. Twelve patients (40%) presented with kidney toxicity during the first-line therapy, the other 18 (60%) were given > 1 previous treatments.

The most frequently administered therapies were immunotherapy (#9 pts, 30%), targeted therapy (#8 pts, 26.7%), immunotherapy plus targeted therapy (#4 pts, 13.3%), immunotherapy plus chemotherapy (#4 pts, 13.3%), targeted therapy plus chemotherapy (#5 pts, 16.7%).

Eleven patients (36.7%) developed AKI, 6 (20%) AKI with isolated proteinuria, 4 (13.3%) AKI with proteinuria and hematuria, 8 (26.7%) isolated proteinuria (in nephrotic range in 5 patients) and 1 (3.3%) proteinuria and hematuria.

In patients receiving first-line immunotherapy before kidney biopsy the average latency time was 5.6 months (min 3 – max 9).

**Figure 2:**
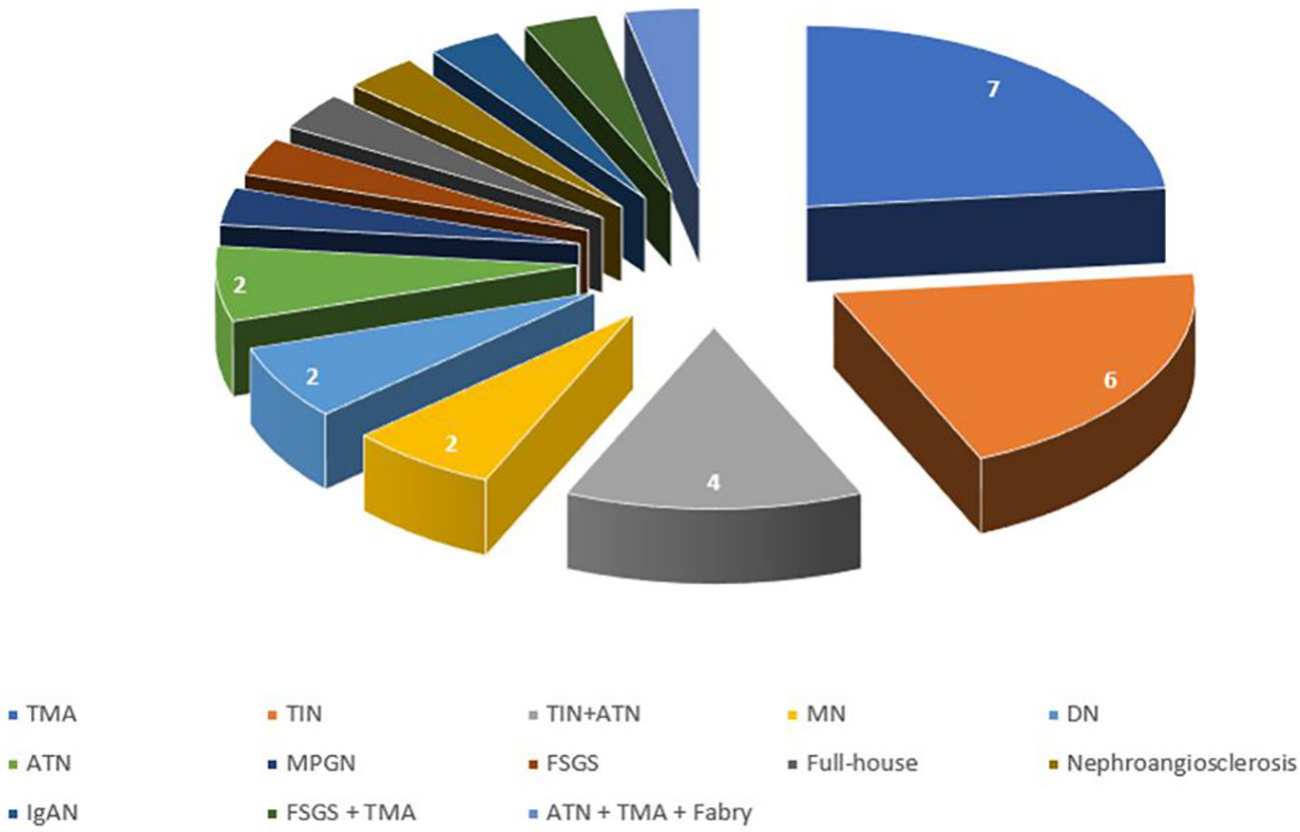
Distribution of histological diagnosis. TMA, thrombotic microangiopathy; TIN, tubule-interstitial nephritis; ATN, acute tubular necrosis; MN, membranous nephropathy; DN, diabetic nephropathy; MPGN, membranoproliferative glomerulonephritis; FSGS, focal segmental glomerulosclerosis; IgAN, IgA nephropathy.

**Figure 3:**
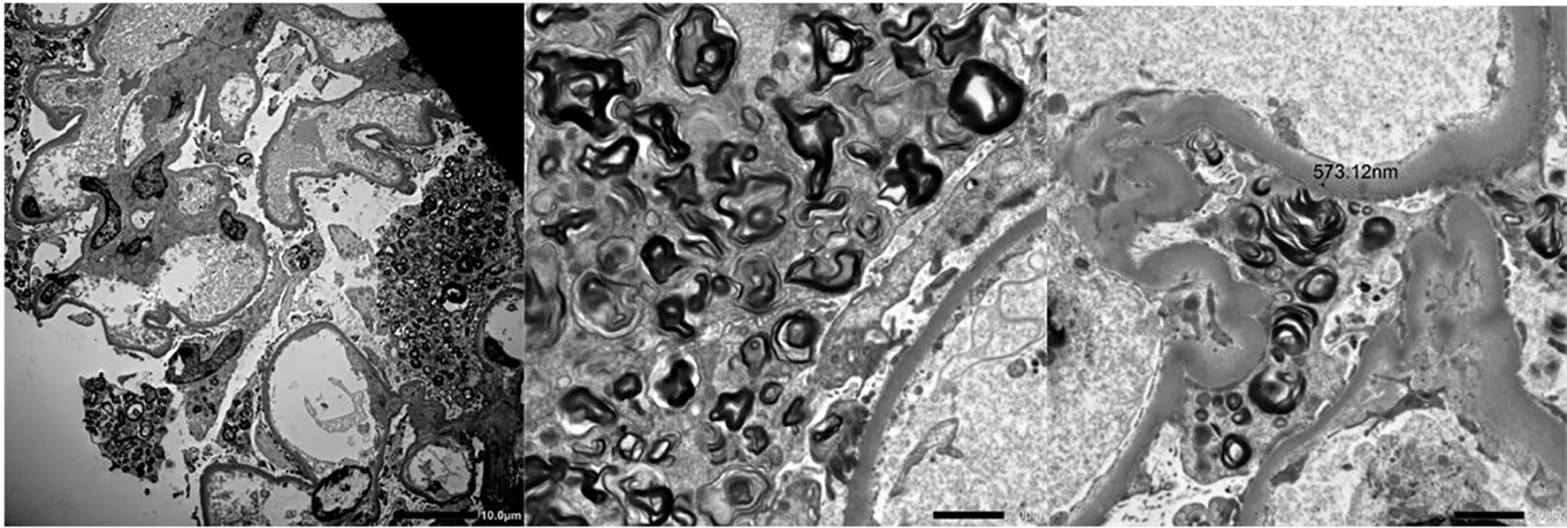
Electron microscopy showing Zebra Bodies in a patient who received a diagnosis of Fabry disease.

The most common disease (**Figure 2**) was TIN (#10 patients, 30%), that was associated with acute tubular necrosis (ATN) in 4 patients, and TMA (#7 patients, 23.3%). Two patients had anti-PLA2r positive MN (6.7%), 2 had diabetic nephropathy (6.7%) and 2 had ATN (6.7%). The other patients had a membranoproliferative glomerulonephritis (MPGN) (1 patient), FSGS (1 patient), full-house nephropathy (1 patient), nephroangiosclerosis (1 patient) and IgAN (1 patient). One patient had a double nephropathy (FSGS plus TMA), and one had 3 different histological injuries (ATN plus TMA plus Fabry Disease) (**Figure 3**).

**Figure 4:**
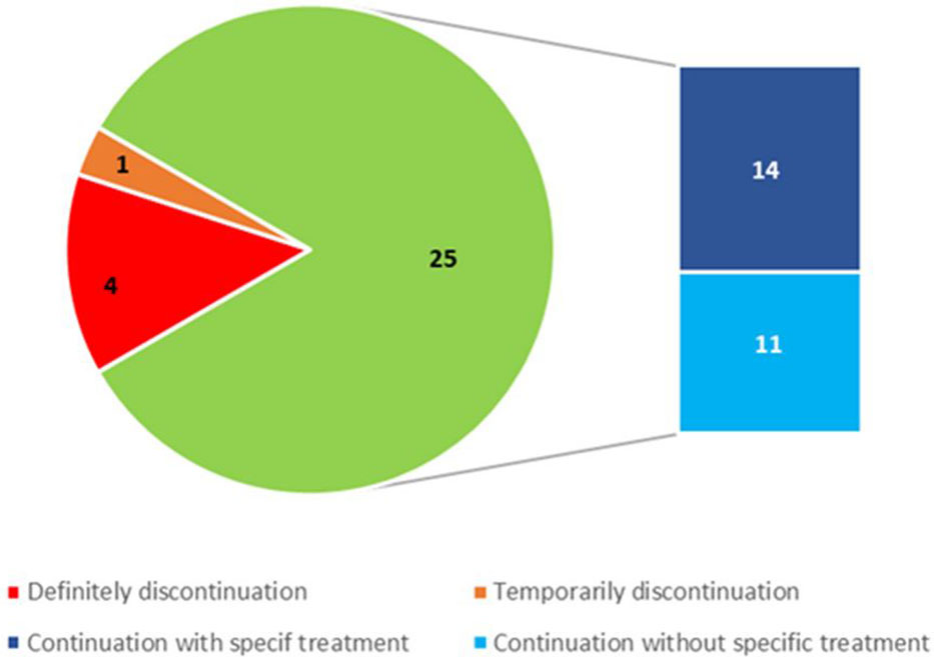
Number of treatment discontinuations or continuations with or without specific therapy after kidney biopsy.

After kidney biopsy, 16 out of the 30 patients were specifically treated according to the histological diagnosis (**Figure 4**). Fourteen patients (46.7%) were treated with steroids: 10 patients with TIN (100% of pts), 1 patient with MN, 1 with MPGN, 1 with full-house glomerulonephritis and 1 with IgAN. All of them received intravenous methylprednisolone pulses followed by oral prednisone that was rapidly tapered and discontinued within 3 months. One patient with MN was treated with low doses of rituximab. The patient with TMA required dialysis that was discontinued following a 3 month eculizumab therapy.

Overall kidney response was obtained in all patients treated with glucocorticoids, while complete kidney response was achieved in the patient treated with rituximab. To date, no patient has experienced a relapse.

Cancer treatment was continued without change in 21 out of 30 patients (70%). One drug was discontinued in 4 out of 30 patients (13.3%) who underwent multiple drug therapy. Treatment was temporarily discontinued in 1 patient (3.4%) and definitively in 4 (13.3%) (**Table 2**). Patients who required permanent discontinuation of treatment had a TMA on kidney biopsy and were on anti-VEGF therapy (3 patients on bevacizumab and 1 patient on sunitinib). In two of them therapy was discontinued during the maintenance regimen.

**Table 2:** Clinical characteristics at the time of kidney biopsy, histological findings and indications for continuation/discontinuation of cancer treatment after kidney biopsy.

Pt	sCr mg/dl	GFR ml/min	uPT g/day	Histological diagnosis	Discontinuation of ongoing treatment
1	1.6	48	0.2	TIN	No
2	1.5	45	3.6	MN (PLA2r+)	No
3	3.6	22	0.3	TIN	No
4	0.9	75	5.4	MPGN	No
5	HD	HD	1.7	TMA	Yes
6	4	14	0.3	TIN + ATN	No
7	2.1	29	1.6	Diabetic Nephropathy	No
8	1.4	45	7.6	FSGS	No
9	0.7	79	1.6	TMA	Partial
10	2.2	36	1.8	TIN (chronic)	No
11	2.6	38	3	ATN	No
12	1.3	50	2.1	TMA	Partial
13	4.1	14	10	Diabetic Nephropathy	No
14	4.1	18	0.5	TIN	No
15	0.8	58	2.7	TMA + FSGS	Partial
16	4.5	14	0.2	TIN + ATN	No
17	2	28	0.2	TIN + ATN	Temporary
18	0.7	82	0.4	TMA	No
19	5.9	20	0.6	Immunocomplex GN (Full House)	No
20	8.7	8	1.2	TIN	No
21	1.1	84	1.6	TMA	Yes
22	0.8	72	3.7	TMA	Yes
23	3.2	23	2.3	Nephroangiosclerosis	No
24	6.2	8	1.5	TMA + ATN + Fabry Disease	Partial
25	HD	HD	0.1	ATN	No
26	2	25	0.6	TIN	No
27	3.1	21	1.3	IgAN	No
28	3.2	18	10	MN (PLA2r +)	No
29	1.8	25	0.4	TIN + ATN	No
30	1.7	27	3.8	TMA	Yes

HD, Hemodialysis; TIN, tubular-interstitial nephritis; MN, membranous nephropathy; membranoproliferative glomerulonephritis; TMA, trombotic microangiopathy; ATN, acute tubular necrosis; FSGS, focal segmental glomerulosclerosis; IgAN, IgA nephropathy.

## Discussion

New cancer therapies have completely changed the concept of therapeutic targets, but have forced clinicians to face new adverse events. Adverse events triggered by these new types of therapy need to be managed differently from adverse events triggered by traditional therapy with the management tailored to the type of kidney insult (31). From a theoretical point of view the ideal target should be essential for malignant cells survival and unexpressed on normal cells, leading to malignant cell death and minimal effect on normal cell function (32). In real life this is not the case. Anti-cancer molecular targeted therapies are associated with side effects involving different organs, including the kidney, with a number of manifestations including AKI (31). In this context, a kidney biopsy is the reliable method to evaluate kidney involvement and provide specific treatment.

As per the recent literature (33, 34), the most frequent indication for kidney biopsy was AKI (>55%) that was isolated or associated with proteinuria with or without hematuria. Our data, in agreement with the literature (35), showed that the most frequently histological feature during immunotherapy was tubulointerstitial nephritis which can occur alone or in combination with other kidney lesions. Vascular injury and glomerular injury occur with anti-angiogenesis drugs targeting the vascular endothelial growth factor (36). Although a number of lesions have been described in association with targeted therapy, TMA is the most common lesion associated with agents targeting vascular endothelial growth factor and it is frequently associated with acute kidney injury (6). In our cohort, kidney biopsy revealed TMA in 8 out of 17 patients (47%) treated with an anti-VEGF drug.

Currently, adverse kidney events result in reduction or discontinuation of therapy in a high percentage of patients, thereby affecting patient outcome. There are no specific evidence-based guidelines for the management of adverse kidney events in patients receiving anti-cancer molecular targeted therapies. The American guidelines recommend temporary discontinuation of bevacizumab in patients with proteinuria >2 g/day, and permanent discontinuation for nephrotic syndrome regardless of the cause. In regards to TKIs discontinuation, FDA guidelines are even more confusing: proteinuria ≥3 g/day for pazopanib, ≥2 g/day for lenvatinib, and undefined proteinuria for axitinib. There are no guidelines for other agents such as sorafenib, sunitinib, vandetanib and cabozantinib. With regard to ICIs, the guidelines of the Society for Immunotherapy and the American Society of Oncologists (37) mention “symptomatic nephritis” and refer to kidney function as estimated on serum creatinine. The intervention of the nephrologist is only foreseen following therapy discontinuation. Definitive discontinuation is indicated in the presence of nephrotic range proteinuria regardless of the cause.

Based on histological findings, definitive discontinuation of treatment in our cohort was indicated in a very limited number of patients (13.3%). All of them had anti-VEGF-related TMA. Treatment discontinuation was unneeded in patients treated with ICIs. In patients treated with multidrug therapy that combined conventional anticancer drugs with molecular targeted agents, the histological findings made it possible to identify the weight of specific injury. That led to targeted modifications of the therapeutic protocols, and allowed discontinuation of a single drug. In patients treated with the “pembrolizumab-axitinib” combination (28), histological evaluation allowed identification of specific lesions and discontinuation of the drug that was actually responsible for the kidney injury.

The optimal treatment for adverse events in patients who undergo new anti-cancer therapies is debated. Glucocorticoids are the cardinal treatment for adverse events. However, dose and treatment duration remain to be defined. Consensus guidelines recommend on occasions the addition of a second immunosuppressant (38–40). In our cohort all patients who underwent a biopsy-addressed treatment achieved kidney response and to date, none experienced a relapse. It is worth to remind that patients who do not achieve kidney recovery had a higher mortality compared to those with complete or even partial kidney remission (33). The increased mortality associated with failure to recover from AKI may reflect the limited therapeutic options offered to patients with impaired kidney function, and emphasized the relevance of early recognition and treatment of kidney impairment before irreversible damage occurs (33).

Notably, histological findings were not consistent with drug-related damage in 6 out of 30 patients (20%). These findings allowed to avoid glucocorticoid prescription which would have been indicated according to current guidelines but would have been useless in these patients. In two patients kidney biopsy revealed double and triple nephropathy, respectively. In the latter case, the patient had a diagnosis of Fabry disease (**Figure 4**) which was confirmed by genetic testing.

In conclusion, the trend of rapid approval of new drugs brings challenges to the management of drug-related kidney toxicities. The clinician’s challenge is allowing patients to continue life-saving treatments by effectively managing adverse kidney effects. Nephrologists and nephro-immunologists should learn to treat the nephrotoxic sequelae of cancer therapy and allow continuation of the life-saving treatment (41). In this context kidney biopsy is a mandatory diagnostic and prognostic tool.

To our knowledge this is one of the single-center studies with the largest number of cancer patients treated with targeted/immunotherapy who systematically underwent histological evaluation after developing AKI or urinary abnormalities. Our data indicate that kidney biopsy should be considered in every cancer patient who develops urinary abnormalities or shows a worsening of kidney function during treatment with immunotherapy or targeted therapy. Based on histological features treatment discontinuation can be limited to a restricted number of patients while allowing to achieve an overall kidney response in patients receiving a specific treatment.

Kidney biopsy is cardinal in the management of kidney toxicities from new cancer agents and in spite of current international guidelines should be encouraged.

## Data availability statement

The original contributions presented in the study are included in the article/supplementary material. Further inquiries can be directed to the corresponding author.

## Ethics statement

Written informed consent was obtained from the patients for the publication of any potentially identifiable data included in this article.

## Author contributions

Author Contributions Conceptualization: RF and DR. Resources: AB and AC. Collection data: MC and GD. Writing original draft preparation: RF and DR. Supervision: SS. All authors contributed to the article and approved the submitted version.
